# A transcriptional co-expression network-based approach to identify prognostic biomarkers in gastric carcinoma

**DOI:** 10.7717/peerj.8504

**Published:** 2020-02-14

**Authors:** Danqi Liu, Boting Zhou, Rangru Liu

**Affiliations:** 1 Department of Pharmacy, Xiangya Hospital, Central South University, Changsha, People’s Republic of China; 2 Institute for Rational and Safe Medication Practices, National Clinical Research Center for Geriatric Disorders, Xiangya Hospital, Central South University, Changsha, People’s Republic of China; 3 Department of Clinical Pharmacology, Xiangya Hospital, Central South University, Changsha, People’s Republic of China; 4 Key Laboratory of Tropical Diseases and Translational Medicine of the Ministry of Education & Hainan Provincial Key Laboratory of Tropical Medicine, Hainan Medical College, Haikou, People’s Republic of China

**Keywords:** Gastric carcinoma, Gene expression profiling, Prognostic biomarker, System biology, WGCNA

## Abstract

**Background:**

Gastric carcinoma is a very diverse disease. The progression of gastric carcinoma is influenced by complicated gene networks. This study aims to investigate the actual and potential prognostic biomarkers related to survival in gastric carcinoma patients to further our understanding of tumor biology.

**Methods:**

A weighted gene co-expression network analysis was performed with a transcriptome dataset to identify networks and hub genes relevant to gastric carcinoma prognosis. Data was obtained from 300 primary gastric carcinomas (GSE62254). A validation dataset (GSE34942 and GSE15459) and TCGA dataset confirmed the results. Gene ontology, the Kyoto Encyclopedia of Genes and Genomes (KEGG) pathway enrichment analysis, and gene set enrichment analysis (GSEA) were performed to identify the clusters responsible for the biological processes and pathways of this disease.

**Results:**

A brown transcriptional module enriched in the organizational process of the extracellular matrix was significantly correlated with overall survival (HR = 1.586, *p* = 0.005, 95% CI [1.149–2.189]) and disease-free survival (HR = 1.544, *p* = 0.008, 95% CI [1.119–2.131]). These observations were confirmed in the validation dataset (HR = 1.664, *p* = 0.006, 95% CI [1.155–2.398] in overall survival). Ten hub genes were identified and confirmed in the validation dataset from this brown module; five key biomarkers (*COL8A1*, *FRMD6*, *TIMP2*, *CNRIP1* and *GPR124 (ADGRA2)*) were identified for further research in microsatellite instability (MSI) and epithelial-tomesenchymal transition (MSS/EMT) gastric carcinoma molecular subtypes. A high expression of these genes indicated a poor prognosis.

**Conclusion:**

A transcriptional co-expression network-based approach was used to identify prognostic biomarkers in gastric carcinoma. This method may have potential for use in personalized therapies, however, large-scale randomized controlled clinical trials and replication experiments are needed before these key biomarkers can be applied clinically.

## Introduction

Gastric carcinoma (GC) is one of the most aggressive and life-threatening malignancies. It ranks as the second-most common cause of tumor-related deaths worldwide, accounting for approximately 10% of all tumor deaths (Song et al., 2014). More than 50% of GC-related deaths occurred in East Asia, specifically in China (Ferlay et al., 2010; Kang et al., 2015). The development of malignant GC is a multi-step process. Patients diagnosed with advanced GC generally have a poor prognosis and the 5-year overall survival (OS) rate is approximately 20% (Catalano et al., 2009). Medical evaluation after gastrectomy and chemotherapy (CT) or chemo-radiotherapy (CRT) in a neo-adjuvant or adjuvant setting for GC is limited and the results of these treatments are disappointing. The lack of precision treatments and assessment strategies has prompted researchers to investigate the oncogenic abnormalities of GC to appraise survival rates and guide medical decisions. Identifying therapeutic targets and prognostic biomarkers for early detection of GC and developing appropriate therapies is a prospective approach for defining the subtypes of GC and improving the prognosis of patients with advanced GC. However, the potential heterogeneities and complexities of GC make it difficult to identify reliable factors for determining effective clinical treatments (Liu et al., 2017).

The complexity of GC is highlighted by its molecular biomarkers. However, the molecular subtyping of GC is based on several commonly used biomarkers (Liu et al., 2017), such as microsatellite instability (MSI) (Cancer Genome Atlas Research Network, 2012) and epithelial-to-mesenchymal transition (EMT) (Loboda et al., 2011). Four new and distinct GC subtypes associated with survival were proposed in a large population-based study (Cristescu et al., 2015).

Microarray technology is the preferred method to investigate the gene expression profiles of GC; enormous amounts of data have been produced to measure the factors that drive GC diagnosis and prognosis. The extraction of transcriptional-based prognostic signatures and clinical outcomes has been studied extensively in a limited number of subtype-related cases (Cho et al., 2011; Deng et al., 2012; Wu et al., 2013; Zouridis et al., 2012). However, there have been no high-grade repeatability of GC-related studies to verify the results. GC is comprised of various molecular entities with different biological processes; thus, the prognostic signatures may be included in disparate GC subtypes making it necessary to study tumorigenesis in different GC entities.

A weighted gene co-expression network analysis (WGCNA) based on gene-associated phenotypes from transcriptomics data can be used to construct functional clusters of co-expressed genes (modules). This relatively novel co-expression approach also allows for investigation of a consistent expression relationship; these modular genes share common biological regulations and pathways (Stuart et al., 2003). WGCNA has been widely used in a variety of diseases, such as breast cancer (Clarke et al., 2013; Liu, Guo & Zhou, 2015), lung cancer (Li et al., 2013; Liu et al., 2015), hepatocellular carcinoma (Pan et al., 2016; Zhang et al., 2017a), glioma cancer (Ivliev, T Hoen & Sergeeva, 2010), head and neck squamous cell carcinoma (Liu et al., 2018), cervical cancer (Ge et al., 2018), bone mineral density (BMD) (Farber, 2010) and coronary artery disease (Liu, Jing & Tu, 2016). Earlier studies involving the WGCNA identified modular biomarkers associated with prognosis and potential therapeutic targets. Horvath et al. (2006) identified the *ASPM* gene as a potential novel molecular target in glioblastoma. Yepes et al. (2016) used WGCNA to discover the miRNAs *100*, *let-7c*, *125b*, and *99a* associated with the diffuse histological subtype; the *let-7* miRNA family was shown to play a central role in regulatory relationships.

In this study, WGCNA was used to analyze a large sample of global transcriptome data from gastric tumors in 300 GC patients. Our research sought to identify the gene modules and hub genes related to GC patient prognosis. Our findings were validated by independent datasets of GC samples from other institutions.

## Materials & Methods

### Available microarray-based mRNA expression datasets and preprocessing

The training dataset used for co-expression construction was composed of 300 primary GC tumor specimens obtained at the time of total or subtotal gastrectomy from Samsung Medical Center, Seoul, Korea, from 2004–2007. This dataset was also part of the Asian Cancer Research Group (ACRG) study. These data were downloaded from the Gene Expression Omnibus (GEO) database using accession number GSE62254 (Cristescu et al., 2015) and comprised the largest set of samples ever downloaded from the database.

The validation dataset was constructed from 248 primary GC samples from the Singapore patient cohort known as the Gastric Cancer Project ’08. This dataset was used to confirm the relationship of gene modules or biomarkers with survival of GC. Raw data with .CEL profiles from two studies were downloaded from GEO using accession numbers GSE34942 and GSE15459 (Ooi et al., 2009); there were 56 and 192 available samples with detailed information, respectively.

**Figure 1:**
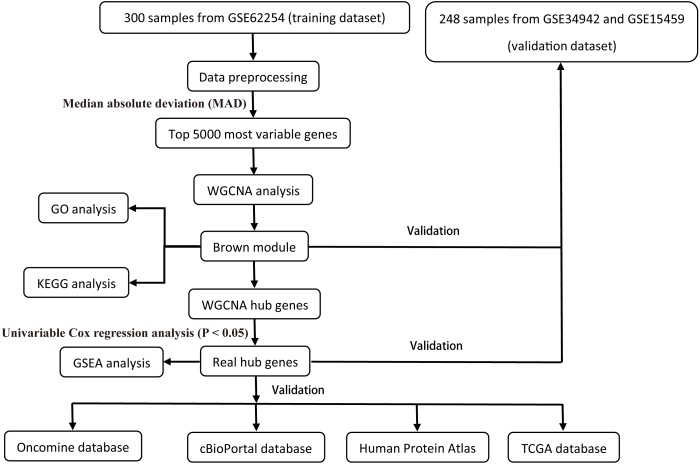
Flow diagram of the study.

All raw expression data was produced using the Affymetrix Human Genome U133 Plus 2.0 Array™ (HG-U133_Plus_2, Affymetrix, Inc., Santa Clara, CA) and normalized with robust multi-array average (RMA) algorithms (Irizarry et al., 2003) using the affy R package (Gautier et al., 2004). The validation dataset was adjusted for potential batch effects among multiple datasets using the ComBat algorithm (Pavlou et al., 2014). Probe sets with available gene symbols were reserved for subsequent analysis and probe-level expression data were transformed into gene-level expression data by merging the probes according to the official annotation file. The average expression values for the multi-probes were calculated as the corresponding gene expression value for one gene. The primary endpoints for the training dataset were overall survival (OS) and disease-free survival (DFS); overall survival (OS) was regarded as the endpoint event for the validation dataset. Gene expression profiles (Illumina HiSeq RNA Seq), level 3 data, and phenotype data of stomach cancer from The Cancer Genome Atlas Project database (TCGA-STAD) were downloaded through the UCSC Xena portal (https://xena.ucsc.edu/) to validate the identified hub genes. All of the gene expression values were in log_2_ (*x* + 1) transformed normalized count for subsequent analysis. Patients chosen for biomarker identification met the following criteria: (1) histologic diagnosis of primary GC; and (2) available RNA expression profiles and complete clinic-pathological and follow-up data. After sample filtering, 374 patients were enrolled for further analysis. The demographics are listed in Table 1. The hub genes were also validated in the cBio Cancer Genomics portal (https://www.cbioportal.org/) (Gao et al., 2013; Cerami et al., 2012). The Human Protein Atlas (http://www.proteinatlas.org) (HPA) was applied to the protein expression dataset to validate the immunohistochemistry of the identified hub genes. Tissues were defined by the normal tissues of stomach cancer and the pathology was defined by the tumor tissues. The selection process of the prognostic biomarkers is shown in Fig. 1.

**Table 1:** Basic characteristics of the datasets.

**Characteristics**	**Training dataset(*n* = 300)**	**Validation dataset(*n* = 248)**	**TCGA dataset(*n* = 374)**
**Gender (%)**			
Male	199 (66.33)	161 (64.92)	242 (64.71)
Female	101 (33.67)	87 (35.08)	132 (35.29)
**Age: mean (sd)**	61.94 (11.36)	65.40 (12.50)	65.11 (10.62)
**Lauren type (%)**			
Intestinal	146 (48.67)	138 (55.65)	–
Diffuse	135 (45)	86 (34.68)	–
Mixed	17 (5.67)	22 (8.87)	–
Unknown	2 (0.67)	2 (0.81)	–
**pStage (%)**			
I	30 (10)	42 (16.94)	52 (13.90)
II	97 (32.33)	40 (16.13)	121 (32.35)
III	96 (32)	91 (36.69)	164 (43.85)
IV	77 (25.67)	73 (29.44)	37 (9.89)
Unknown	0 (0.00)	2 (0.81)	0
**Mol. Subtype (%)**			
MSS/TP53^−^	107 (35.67)	–	–
MSS/TP53^+^	79 (26.33)	–	–
MSI	68 (22.67)	–	–
MSS/EMT	46 (15.33)	–	–
**OS**			
Time mean (sd)	50.60 (31.42)	38.81 (42.69)	20.40 (17.93)
Event (%)	152	122	146
**DFS**			
Time mean (sd)	33.72 (29.82)	–	–
Event (%)	152	–	–

**Notes.**

Overall survival (OS), Disease-free survival (DFS).

### Gastric carcinoma molecular subtypes

GC patients were divided into four groups according to their molecular subtypes as described by Cristescu et al. (2015). The molecular subtype signatures had the following tumor biomarkers: MSI (microsatellite instability); MSS/EMT (epithelial-tomesenchymal transition); MSS/TP53^+^ (the tumor protein 53 (TP53)-active); and MSS/TP53^−^ (the tumor protein 53 (TP53)-inactive). Information on the molecular subtypes can be found in the supplement materials of the original publication (Cristescu et al., 2015). In this dataset, 68 samples were classified as MSI, 46 samples were classified as MSS/EMT, 79 samples were classified as MSS/TP53^+^, and 107 samples were classified as MSS/TP53^−^. A subsequent survival analysis according to the molecular subtype was not performed in this dataset because the validation dataset lacked information regarding those subtypes.

### Weighted gene co-expression network detection

The training dataset (GSE62254) was the target for WGCNA using the “wgcna” R package (Langfelder & Horvath, 2008). The top 5000 most variable genes were selected according to the median absolute deviation (MAD), which restricted the analysis to those genes with a notable variance in expression; MAD is a robust measure of variability for the construction of co-expression networks.

Pearson’s correlation coefficient (PCC) was used to assess the relationship between each pair of the 5000 genes. These data were used to construct an unsupervised co-expression-based adjacency matrix with a soft threshold power of 4 based on the scale-free topology criterion to raise the matrix to simulate a realistic network structure (Zhang & Horvath, 2005). The intramodular connectivity (k.in) measured how connected, or co-expressed, a given gene was with respect to the genes of a particular module. The connection strengths were assessed by calculating the topology overlap (TO) (Yip & Horvath, 2007), and the modules were defined as sets of genes with a high TO (Zhang & Horvath, 2005). The topological overlap matrix (TOM) was the network distance measure for each gene pair from the adjacent matrix. A TOM-based dissimilarity measure (1-TOM) was utilized to achieve an average hierarchical linkage clustering (Ravasz et al., 2002). Gene modules were defined using a dynamic hybrid branch-cutting algorithm with a cutoff of 0.95 and a minimum module size and cutoff of 30 in the hierarchical clustering dendrogram on the basis of TOM dissimilarity (Langfelder, Zhang & Horvath, 2008). The module eigengene (ME) was calculated by a principal component analysis (PCA) by defining the first principal component of a given module. The module membership, also known as eigengene-based connectivity kME, related each gene expression profile with the ME of a specific module. The MEs of a summary profile were used to assess the underlying correlation of gene modules with the clinico-pathological variables and survival.

### Survival analysis and identification of hub genes

Survival analysis was performed using the survival R package with the hazard ratio (HR) and its corresponding 95% confidence interval (CI) determined by the Cox regression module and Kaplan–Meier survival curves (http://cran.r-project.org/web/packages/survival/index.html). OS or DFS were considered to be the survival endpoints. Covariates, including the Lauren’s diffuse type and intestinal type, tumor type, stage and molecular type were corrected via multivariate analysis to estimate the prognostic effects of modules and hub genes. For modules or single gene-based associations, each ME or gene expression value was a continuous variable that was categorized as having a high or low expression according to the median expression value at the cutoff point. For a given ME/gene, the patients were split into two groups known as high expression (≥median expression of the ME/gene) and low expression (<median expression of the ME/gene).

The gene significance (GS) was defined as minus log 10 of the univariate Cox proportional hazard-regression *p*-values in the single gene-based analysis. Hub genes, namely, highly connected genes, were genes that tended to have high network connectivity (k.in) that determined the connection strength (co-expression) of a specific gene with other genes in a given module (Liu et al., 2017). Genes that satisfied the following criteria were classified as hub genes: (i) GS > 2; (ii) targeted module kME value >0.85.

### Functional annotation of the targeted module

Gene enrichment analysis of the categorical biological processes of gene ontology (GO) was conducted on the targeted modules associated with GC patient survival to explore further insights into the genes via the DAVID annotation tool (http://david.abcc.ncifcrf.gov/) (Dennis Jr et al., 2003). The fold enrichment was calculated for all GO terms in the given ontologies to examine the enrichment degree of the specific genes for all genes on the array. Multiple tests were performed with *p*-values adjusted according to the Bonferroni, Benjamini and false discovery rate (FDR) methods.

The Kyoto Encyclopedia of Genes and Genomes (KEGG) pathway enrichment analysis for genes in the targeted module was conducted via the ClueGO (Bindea et al., 2009) and CluePedia (Bindea, Galon & Mlecnik, 2013) applications in the Cytoscape v3.3.0 software. Only terms with *p*-values <0.05 were retained.

### Gene set enrichment analysis (GSEA)

To obtain further insight into the potential mechanisms of hub genes significantly associated with survival, GSEA (http://software.broadinstitute.org/gsea/index.jsp) (Subramanian, Tamayo & Mootha, 2005) was conducted based on the median expression level of the significant hub genes to map for the KEGG pathways database. The annotated gene set *c2.cp.kegg.v6.1.symbols.gmt* was chosen as the reference gene set. Differences with a false discovery rate (FDR) of less than 5% had statistical significance; FDR was calculated using the p.adjust function.

## Results

### Detection of gene co-expression modules

**Figure 2:**
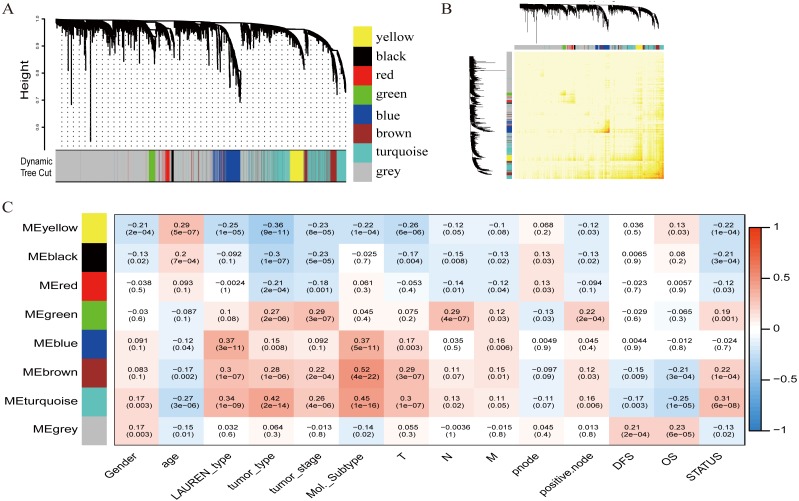
Identification of specific modules defined by WGCNA in the training dataset GSE62254 with 300 primary gastric cancer samples. (A) Hierarchical clustering tree (dendrogram) of median absolute deviation (MAD) genes clustered based on a dissimilarity measure (1-TOM). Each leaf, represented as a short vertical line, corresponds to a gene, and branches of the dendrogram that group together represent densely interconnected, highly co-expressed genes. In total, seven modules ranging from 43 to 1,059 genes in size were identified with each gene color-coded based on module assignment, and gray is reserved for unassigned genes. (B) Topological overlap matrix plot. Genes in the rows and columns are sorted by the clustering tree in (A). (C) Pearson’s correlation coefficient (PCC) matrix among MEs, clinico-pathological variables and survival. Depending on the strength of the correlation, the PCC values range from −1 to +1. A negative value suggests that the genes within a module increase as the variable decreases, whereas the opposite is true if the PCC value is positive.

To investigate the potential patterns of expression among the 5000 most varied genes; WGCNA was conducted on a public microarray-based GC dataset derived from 300 primary GC tumor tissues. Seven informative modules were identified with lengths of 43 to 1,059 genes (Fig. 2A). Each module was assigned a unique color (Table 2), and the gray module, with 2708 non-co-expressed genes, was assigned a gradient color. The topological overlap matrix was plotted according to the expression levels of all genes (Fig. 2B). The MEs and MM (kME) were calculated across all samples and all genes, respectively. The affiliations of all 5000 genes and the complete list of network indices (kME and k.in) for each gene are presented in Data S1.

### Correlation of modules with clinico-pathological variables

To measure the association between the seven identified co-expression modules and clinico-pathological variables, we calculated the PCCs between MEs as continuous variables; gender, age, Lauren’s diffuse type and intestinal type, tumor type, tumor stage, molecular subtype, T (Tumor), N (Node), M (Metastasis), node count, positive node, DFS, OS, and status were also calculated (Fig. 2C). The yellow, brown, and turquoise modules yielded significant associations with Lauren’s type (yellow = −0.25, brown = +0.3, turquoise = +0.34), tumor type (yellow = −0.36, brown = +0.28, turquoise = +0.42), tumor stage (yellow = −0.23, brown = +0.22, turquoise = +0.26), and molecular subtype (yellow = −0.22, brown = +0.52, turquoise = +0.45).

### Gene modules significantly correlated with survival

**Figure 3:**
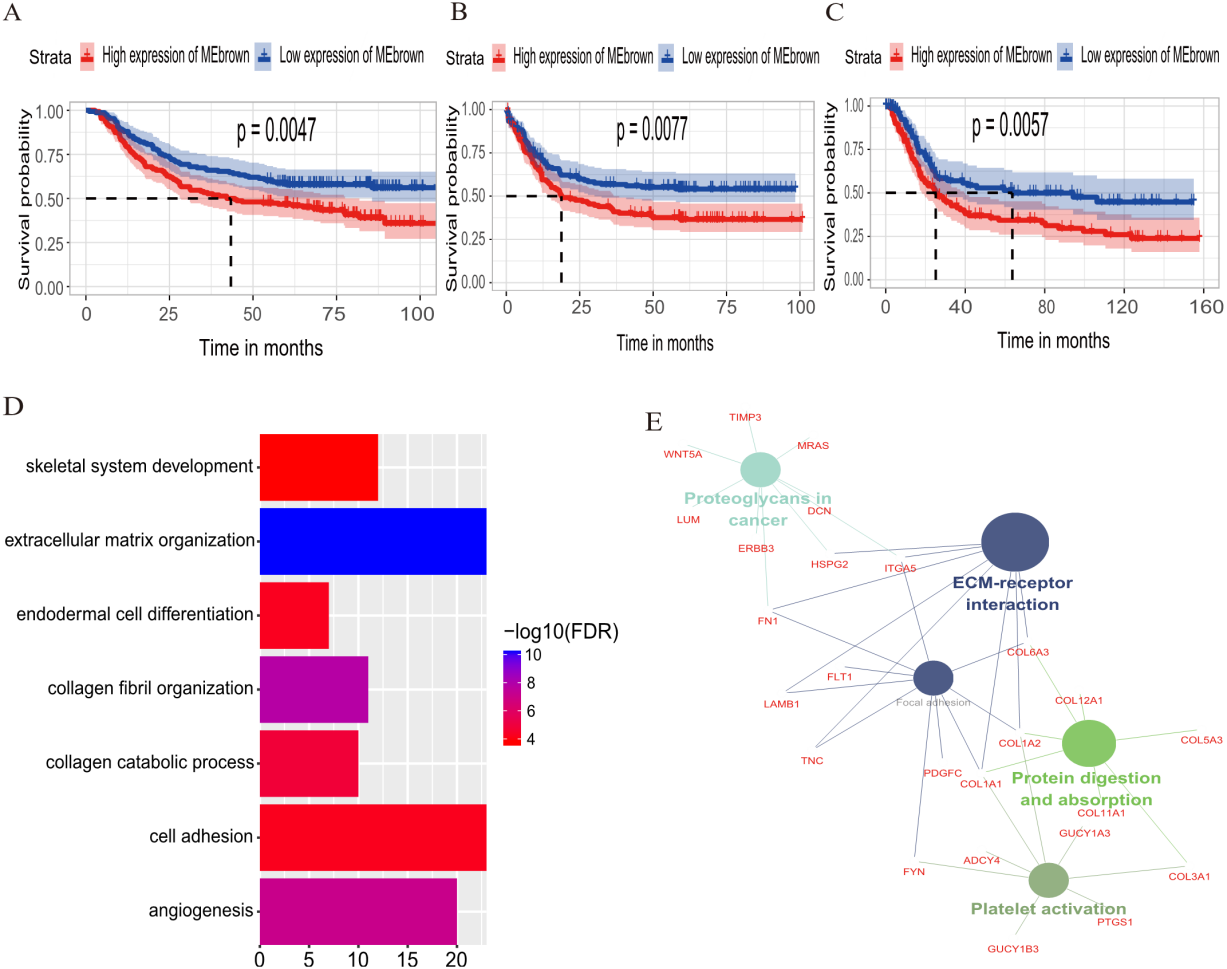
Identification of module brown in the training set and confirmed in the validation set. (A) Relationship between overall survival (OS) and the ME_brown_ in the training dataset. Kaplan–Meier survival plots for overall survival are shown (gastric cancer patients were grouped by the median of module eigengene). Increased expression of the ME_brown_ indicated low overall survival (A) and poor disease-free survival (B). Additionally, the relationship between overall survival (OS) and the ME_brown_ in the validation dataset was presented in (C). The dashed line in K-M curves mean the median survival. It defined that the survival time corresponding to a cumulative survival rate of 0.5 means that only 50% of individuals can live this time. (D) GO enrichment analysis for the 342 genes comprising the brown module identified multiple biological processes with FDR < 0.05. The raw significance output from DAVID was transformed into “−log 10 (FDR)” for plotting. (E) The relationship of KEGG enrichment pathways with *p*-value < 0.05 for the 342 genes involved in the brown module.

The relationship between OS/DFS and modules was assessed as a whole using Cox regression analysis with HRs and corresponding *p*-values (Table 2). The black, brown, green, turquoise, and yellow modules showed significant correlations with OS in the training dataset (module brown: HR = 1.586, *p* = 0.005, 95% CI [1.149–2.189]). However, only the brown module was confirmed in the validation dataset (HR = 1.664, *p* = 0.006, 95% CI [1.155–2.398]). We also found that ME_black_, ME_brown_, ME_green_, and ME_turquoise_ were significantly correlated with DFS in the training dataset. The brown module was the focus in subsequent analyses as its high expression demonstrated poor prognosis in the training (Figs. 3A and 3B) and validation datasets (Fig. 3C).

**Table 2:** Association of expression modules with OS/DFS in the training and validation dataset.

**Modules**	**Gene count**	**Training dataset (*n* = 300)**						**Validation dataset (*n* = 248)**		
		**OS**			**DFS**			**OS**		
		**HR**	**95% CI**	***p*-value**	**HR**	**95% CI**	***p*-value**	**HR**	**95% CI**	***p*-value**
ME_black_	43	0.633	0.458–0.876	0.006^**^	0.695	0.503–0.961	0.028^*^	1.486	1.04–2.121	0.029^*^
ME_blue_	404	0.992	0.721–1.363	0.959	0.991	0.721–1.362	0.955	1.158	0.809–1.657	0.422
ME_brown_	342	1.586	1.149–2.189	0.005^**^	1.544	1.119–2.131	0.008^**^	1.664	1.155–2.398	0.006^**^
ME_green_	113	1.691	1.222–2.34	0.002^**^	1.609	1.163–2.226	0.004^**^	1.105	0.774–1.578	0.583
ME_red_	95	0.756	0.548–1.042	0.088	0.791	0.574–1.09	0.152	1.2	0.84–1.713	0.317
ME_turquoise_	1,059	1.75	1.264–2.424	0.001^***^	1.66	1.198–2.299	0.002^**^	1.208	0.843–1.729	0.303
ME_yellow_	236	0.654	0.474–0.903	0.010^**^	0.749	0.543–1.034	0.079	1.201	0.842–1.713	0.313

**Notes.**

* *p* ≤ 0.05.

** *p* ≤ 0.01.

*** *p* ≤ 0.001.

Overall survival (OS), Disease-free survival (DFS). Hazard ratios (HRs), 95% confidence intervals (CI), and *p*-values were calculated using Cox proportional hazards regression analysis after grouped the gastric cancer patients by the median of gene level.

### Biological insights into the brown module

To illuminate the potential biological insights into the brown module, we performed GO biological process enrichment analysis with DAVID and KEGG pathway analyses using Cytoscape v3.3.0. Seven GO terms were identified that were significantly enriched with FDR < 0.05 (Fig. 3D) and five pathways with FDR < 0.05 (Fig. 3E). The most significant terms from the GO analysis were “extracellular matrix organization” (raw *p*-value = 6.29 × 10^−13^, Bonferroni-adjusted *p*-value = 9.68 × 10^−10^, FDR = 8.11 × 10^−11^) and “ECM-receptor interaction” (raw *p*-value = 2.40 × 10^−5^, Bonferroni-adjusted *p*-value = 3.40 × 10^−4^, FDR = 1.20 × 10^−4^) from the KEGG analysis. A full list of biological GO terms and the KEGG analysis of all co-expression genes in the brown module is shown in Data S2 and S3, respectively.

### Identification and validation of survival-related hub genes

**Figure 4:**
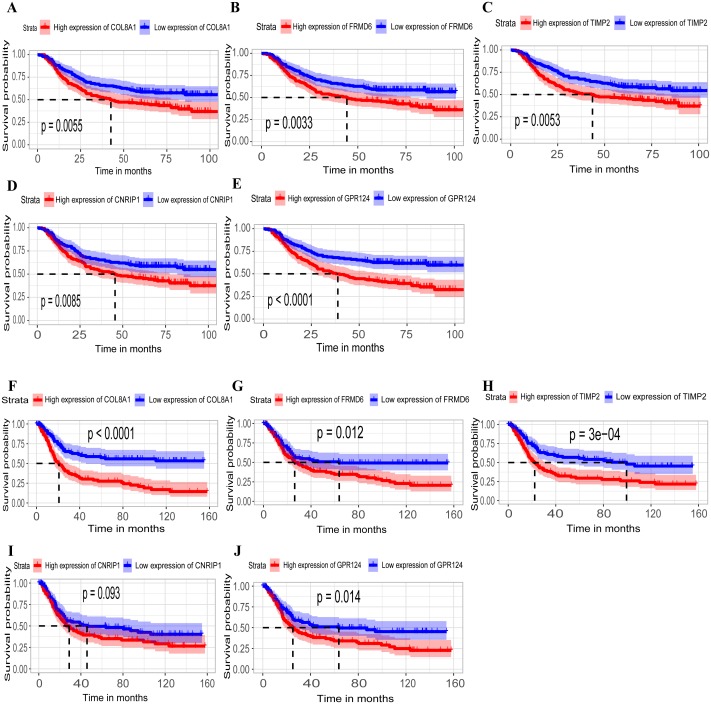
Identification of hub genes in the training set and confirmed in the validation set. (A–E) Relationship between overall survival (OS) and the five key hub genes (COL8A1, FRMD6, TIMP2, CNRIP1, GPR124) in the training dataset. Kaplan-Meier survival curve plots for OS are shown (gastric cancer patients were grouped by the median of the expression level of hub genes). Increased expression of the five genes indicated poor overall survival. Additionally, the relationship between OS and the five key hub genes in the validation dataset was presented in (F–J). Red lines represent high expression of the real hub genes and blue lines represent low expression. The “+” symbol in the panel indicated censored data. The dashed line in KM curves mean the median survival. It defined that the survival time corresponding to a cumulative survival rate of 0.5 means that only 50% of individuals can live this time.

**Figure 5:**
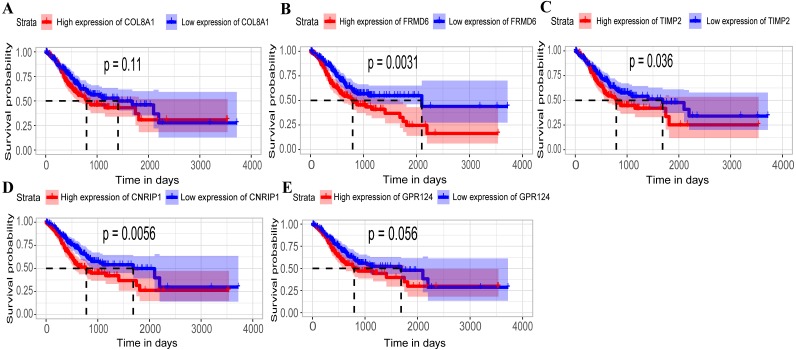
Relationship between overall survival (OS) and the five key hub genes in the TCGA dataset. (A) COL8A1; (B) FRMD6; (C) TIMP2; (D) CNRIP1; (E) GPR124. Kaplan–Meier survival curve plots for OS are shown (gastric cancer patients were grouped by the median of the expression level of hub genes). Increased expression of the five genes indicated poor overall survival. Red lines represent high expression of the real hub genes and blue lines represent low expression. The “+” symbol in the panel indicated censored data. The dashed line in KM curves mean the median survival. It defined that the survival time corresponding to a cumulative survival rate of 0.5 means that only 50% of individuals can live this time.

**Figure 6:**
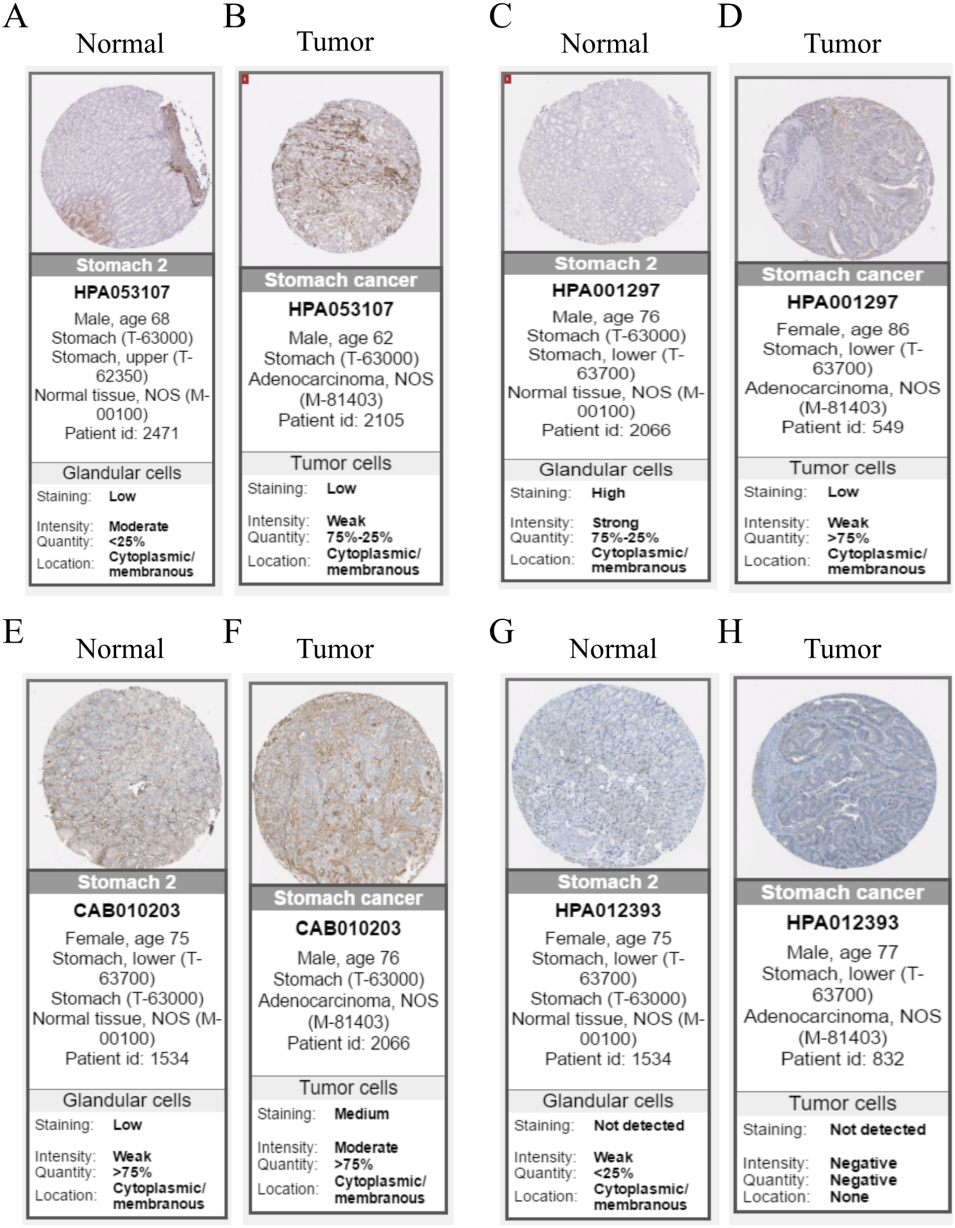
Immunohistochemistry of the five hub genes based on the Human Protein Atlas. There was no related IHC samples of CNRIP1 in the database. (A) Protein levels of COL8A1 in normal tissue. (B) Protein levels of COL8A1 in tumor tissue. (C) Protein levels of FRMD6 in normal tissue. (D) Protein levels of FRMD6 in tumor tissue. (E) Protein levels of TIMP2 in normal tissue. (F) Protein levels of TIMP2 in tumor tissue. (G) Proteins level of GPR124 in normal tissue. (H) Protein levels of GPR124 in tumor tissue.

**Figure 7:**
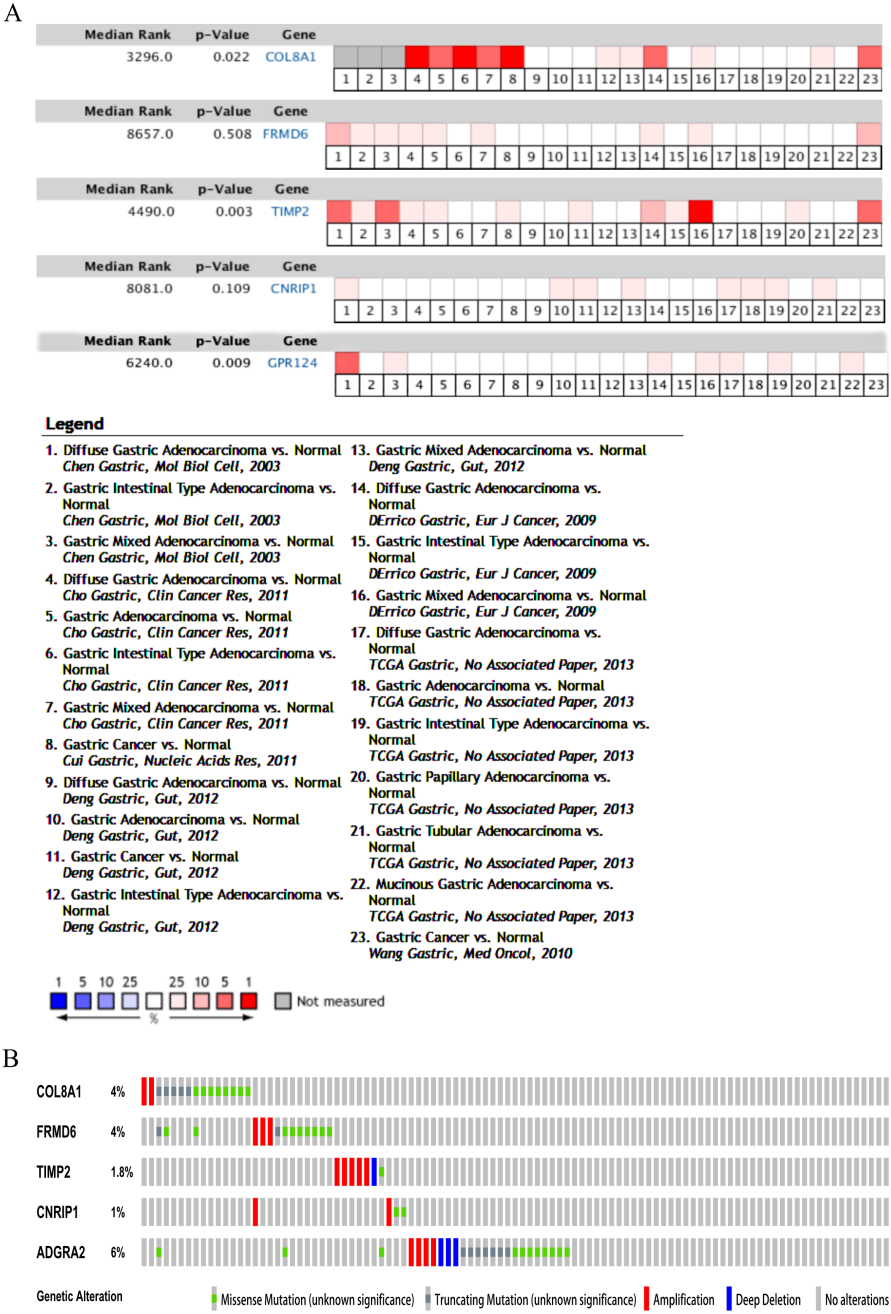
Validation of the five hub genes in Oncomine database across 23 studies (A) and exploration of genetic alterations of the five hub genes via cBioPortal tool (B).

Hub genes are highly likely to serve as key factors in a given module. Ten hub genes were identified from the 342 co-expression genes in the brown module (*COL8A1*, *FRMD6*, *DDR2*, *LOC100505881*, *TIMP2*, *CNRIP1*, *CLEC11A*, *MRC2*, *BGN*, and *GPR124*). These were associated with OS/DFS in the training dataset and were confirmed in the validation dataset with the exception of the relationship between *CNRIP1*, and OS (Table 3). Survival analysis of the hub genes *COL8A1*, *FRMD6*, *TIMP2*, *CNRIP1*, and *GPR124* in the training dataset and validation dataset is shown in Fig. 4. The high expression of the five hub genes presented with poor overall survival and was validated in the TCGA dataset (Fig. 5). We also investigated the hub genes in other modules based on the above screening criterion. The results are presented in Data S1 with highlights. There were no hub genes in the blue, red, and gray modules. Immunohistochemistry (IHC) staining obtained from the Human Protein Atlas database also demonstrated the expression status of hub genes (Fig. 6). There were no related IHC samples of *CNRIP1* in the database. The Oncomine database was used in our analysis and the mRNA levels of *COL8A1*, *TIMP2*, and *GPR124* were higher in tumor tissues compared with normal tissues (Fig. 7A). The OncoPrint module in cBioPortal, an online tool, was used to analyze genetic alterations, including missense mutations, truncating mutations, amplifications and deep deletions (Fig. 7B).

**Table 3:** Relationships between hub genes in module brown with OS/DFS in the training and validation datasets.

**Gene**	**Training dataset (*n* = 300)**						**Validation dataset (*n* = 248)**		
	**OS**			**DFS**			**OS**		
	**HR**	**95% CI**	***p*-value**	**HR**	**95% CI**	***p*-value**	**HR**	**95% CI**	***p*-value**
COL8A1	1.573	1.139–2.171	0.006^**^	1.468	1.063–2.026	0.020^*^	2.5	1.72–3.632	0.000^***^
FRMD6	1.615	1.17–2.231	0.004^**^	1.557	1.127–2.15	0.007^**^	1.591	1.104–2.292	0.013^*^
DDR2	1.612	1.167–2.226	0.004^**^	1.521	1.101–2.1	0.011^*^	1.71	1.187–2.463	0.004^**^
LOC100505881	1.549	1.122–2.138	0.008^**^	1.477	1.07–2.039	0.018^*^	1.792	1.241–2.589	0.002^**^
TIMP2	1.574	1.141–2.171	0.006^**^	1.446	1.049–1.995	0.024^*^	1.943	1.347–2.803	0.000^***^
CNRIP1	1.537	1.113–2.121	0.009^**^	1.457	1.056–2.01	0.022^*^	1.36	0.949–1.948	0.094
CLEC11A	1.706	1.235–2.356	0.001^***^	1.667	1.207–2.303	0.002^**^	1.44	1.004–2.065	0.047^*^
MRC2	1.652	1.194–2.284	0.002^**^	1.543	1.117–2.133	0.009^**^	1.599	1.112–2.3	0.011^*^
BGN	1.613	1.168–2.229	0.004^**^	1.564	1.132–2.16	0.007^**^	1.978	1.368–2.858	0.000^***^
GPR124	1.929	1.39–2.677	0.000^***^	1.878	1.353-2.606	0.000^***^	1.572	1.094–2.259	0.014^*^

**Notes.**

* *p* ≤ 0.05.

** *p* ≤ 0.01.

*** *p* ≤ 0.001.

Overall survival (OS), Disease-free survival (DFS). Hazard ratios (HRs), 95% confidence intervals (CI), and *p*-values were calculated using Cox proportional hazards regression analysis after grouped the gastric cancer patients by the median of gene level.

### Identification of gene modules and hub genes significantly associated with GC subtype-specific survival

The relationship between the gene modules or hub genes and GC molecular subtypes was explored and HRs and accompanying *p*-values were calculated to denote their significance. Analysis revealed that ME_green_ (HR = 1.765, 95% CI [1.043–2.99], *p*-value = 0.034) and ME_turquoise_ (HR = 2.485, 95% CI [1.205–5.124], *p*-value = 0.014) involving 113 and 1059 genes, respectively, were associated with poor OS outcomes within the MSS/TP53^−^ and MSS/EMT molecular subtypes (Table 4). The increased expression of genes in module turquoise indicated poor DFS prognosis in the MSS/EMT molecular subtype (HR = 2.467, 95% CI [1.198–5.081], *p*-value = 0.014) (Table S1).

**Figure 8:**
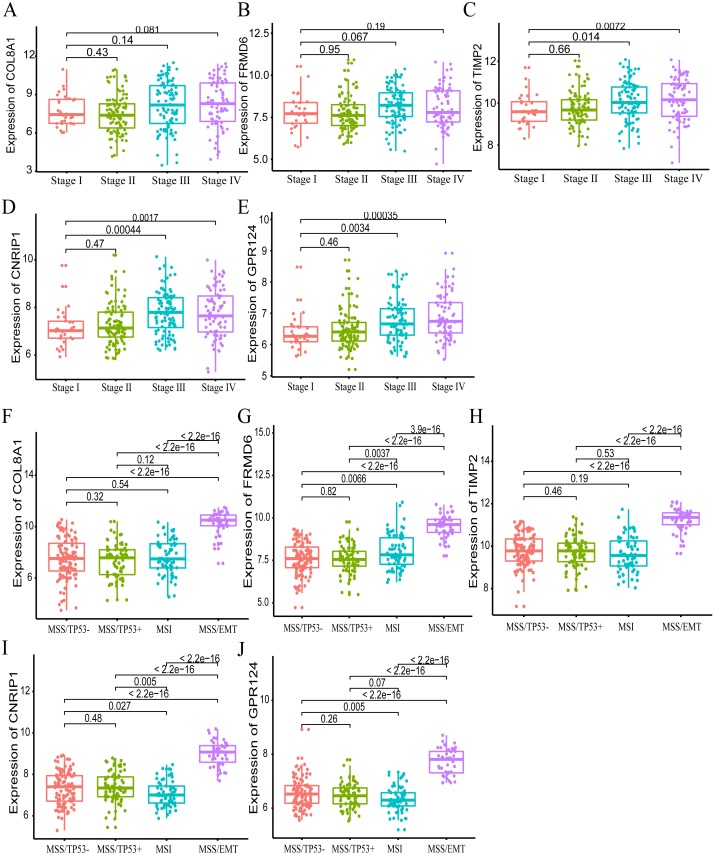
The correlation of the five hub gene expressions with pathological stage (A–E) and molecular subtypes (F–J) in the training dataset. *T*-test was used to evaluate the statistical significance of differences.

Furthermore, we also investigated whether significant correlations could be detected among the 10 hub genes in the brown module and the molecular subtypes in the training dataset. Association analysis revealed that the expression levels of genes *FRMD6* (HR = 2.822, 95% CI [1.156–6.888], *p*-value = 0.023), *TIMP2* (HR = 2.942, 95% CI [1.199–7.217], *p*-value = 0.018), *CNRIP1* (HR = 2.626, 95% CI [1.077–6.401], *p*-value = 0.034) and *GPR124* (HR = 3.696, 95% CI [1.451–9.413], *p*-value = 0.006) were significantly associated with OS in the MSI molecular subtype. The increased expression of the *COL8A1* gene showed poor prognosis (HR = 2.216, 95% CI [1.104–4.447], *p*-value = 0.025) in the MSS/EMT molecular subtype (Table 5). We also found that the *FRMD6* (HR = 2.715, 95% CI [1.116–6.605], *p*-value = 0.028), *TIMP2* (HR = 2.964, 95% CI [1.217–7.217], *p*-value = 0.017), *CNRIP1* (HR = 2.942, 95% CI [1.207–7.168], *p*-value = 0.018), *MRC2* (HR = 2.379, 95% CI [1.007–5.62], *p*-value = 0.048) and *GPR124* (HR = 3.814, 95% CI [1.5–9.694], *p*-value = 0.005) genes were significantly associated with DFS in the MSI molecular subtype. In addition, *COL8A1* gene expression was associated with DFS prognosis in the MSS/EMT molecular subtype (HR = 2.295, 95% CI [1.144–4.602], *p*-value = 0.019) (Table S2). There were significant differences noted in the expression of five hub genes when the tumor stages and molecular subtypes were compared (Figs. 8A and 8B).

### GSEA analysis of key hub genes significantly correlated with subtype-specific survival of GC patients

**Figure 9:**
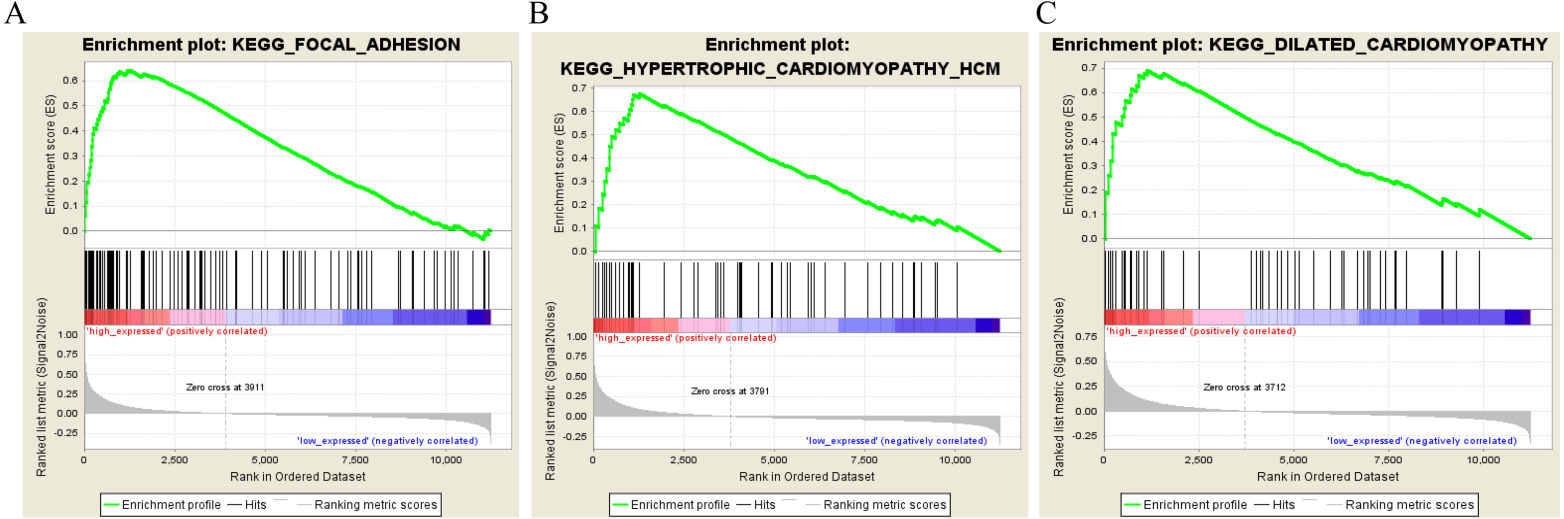
Gene set enrichment analysis of the five hub genes in the brown module. Pathway enriched in the focal adhesion (A), hypertrophic cardiomyopathy HCM (B) and dilated cardiomyopathy (C).

To characterize the potential function of the real hub genes, 300 GC samples were divided into two groups (high vs. low) according to the median expression values of the above hub genes. GSEA was performed based on the expressions of *COL8A1*, *FRMD6*, *TIMP2*, *CNRIP1* and *GPR124*. GC samples in the *COL8A1* and *CNRIP1* high expression group were significantly enriched for focal adhesion (Tables S3–S6). GC samples in the *FRMD6* and *TIMP2* high expression group were significantly enriched for hypertrophic cardiomyopathy (HCM) (Fig. 9; Tables S4–S5). GC samples in the *GPR124* high expression group were significantly enriched for dilated cardiomyopathy (Fig. 9; Table S7).

## Discussion

In this study, WGCNA, which is a systems biology approach, was utilized to research one available mRNA expression dataset composed of 300 primary GC patients to investigate the clusters (modules) and single genes associated with prognostic indicators. The results were confirmed in an independent validation dataset. Compared to select genes that focus on traditional differential expression, WGCNA uses almost 10,000 of the most variable genes to identify the set of genes of interest and conducts a significant association analysis with the phenotype. It makes full use of clinical information and converts thousands of genes and phenotypes into several gene sets and phenotypes, eliminating the problem of multiple hypothesis testing.

Eight distinct gene modules were identified from the top 5000 most variable genes that were filtered by the median absolute deviation (MAD) pre-filtering standard for the co-expression network. Increased expression of the brown module, including 342 genes enriched in an extracellular matrix organization, was associated with positive OS prognosis in the training dataset; this was confirmed in the validation dataset. Furthermore, 10 hub genes were explored as potential biomarkers for GC prognosis in the training dataset and all but *CNRIP1* were confirmed in the validation dataset. Increased expression of the *COL8A1*, *FRMD6*, *TIMP2*, *CNRIP1*, and *GPR124* genes indicated poor survival in the MSI (*n* = 68) and MSS/EMT (*n* = 46) molecular subtypes versus the MSS/TP53^−^ (*n* = 107) and MSS/TP53^+^ (*n* = 79) molecular subtypes. Significant results were achieved in the small sample size, but there may be bias in a larger sample size.

Several hub genes were identified as potential novel markers. Collagen type VIII alpha 1 chain (*COL8A1*), encodes one of the two alpha chains of type VIII collagen. Wang, Chen & Wang (2017) constructed a 9-gene model and *COL8A1* was identified as one of the 7 positive prognostic biomarkers in GC. *COL8A1* is also involved in cell-substrate adhesion and was reported to be significantly down-regulated in non-malignant breast cells (Chen et al., 2014). *COL8A1* may be involved in the proliferation, adhesion, and migration of a variety of cells. The overexpression of *COL8A1* is detected in several rapidly proliferating cells, such as in epithelial and tumor cells (Xu et al., 2001; Bendeck et al., 1996; Paulus et al., 1991). It has been reported that the down-regulation of *COL8A1* may inhibit the proliferation and colony formation of hepatocarcinoma cells (Zhao et al., 2009). This might provide a new potential target for the treatment of hepatocarcinoma. However, the role of *COL8A1* in human cancers, especially in GC, should be further studied. FERM domain containing 6 (*FRMD6*), a protein-coding gene, has been shown to have tumor-related functions and may have tumor suppressor properties in human cancer cell lines (Visser-Grieve, Hao & Yang, 2012). Proteins encoded by *FRMD6* can activate the Hippo kinase pathway, which is an important regulator of cancer development in mammals (Angus et al., 2012; Pan, 2010; Zeng & Hong, 2008). However, few studies have investigated the relationship between this gene and cancer prognosis in humans. TIMP metallopeptidase inhibitor 2 (*TIMP2*), is thought to be a protective factor, and its expression indicates a favorable prognosis in patients with non-small cell lung cancer (NSCLC) in a meta-analysis (Zhu et al., 2015). *TIMP2* expression by cancer-associated fibroblasts (CAFs) was the most potent independent prognostic factor for predicting the clinical outcome of patients in breast cancer (Eiro et al., 2015). Yang et al. (2011) developed a novel CRAd (Ad5/3-CXCR4-TIMP2) for ovarian cancer therapy. Cannabinoid receptor interacting protein 1 (*CNRIP1*), encodes a protein that interacts with the C-terminal tail of cannabinoid receptor 1. Cannabinoid receptor 1 can be found in several tissues, including those of the cardiovascular system, lung, small intestine, peripheral tissues like fat tissue, skeletal muscle, uterus, testes (Russo & De Azevedo, 2019). A study conducted on the *CNRIP1* gene indicated that the *CNRIP1* promoter region may have some value in the early detection and prognostic evaluation of colorectal cancers (Zhang et al., 2017b). This gene may be a potential biological marker of human cancers. G protein-coupled receptor 124 (*GPR124*), was a direct target of *miR-138-5p*, specifically the adhesion G protein-coupled receptor A2 (*ADGRA2*). It has been demonstrated that the expression of *GPR124* in protein and mRNA can be suppressed by *miR-138-5p* in non-small cell lung cancer (NSCLC) cells (Gao et al., 2014).

The classification method in this study is based on the research by Cristescu et al. (2015) for GC molecular subtypes that encompass tumorous heterogeneity. We concluded that the MSI subtype had a better prognosis, similar to the result of Cristescu’s research. We also identified more hub genes that were correlated with the prognosis of the entire population or with molecular subtypes. However, further research is needed to identify accurate outcomes related to the prognostic biomarkers and applications of these genes for safer and more efficient clinical therapies.

Our study has several limitations. Due to the lack of relevant information, such as the molecular subtype in the validation dataset, the results of the training dataset could not be confirmed. As a retrospective study, the patient cohort was heterogeneous, and the significance and robustness of the results and hub genes in the prognostic assessment must be validated in prospective patient cohorts. Lastly, although WGCNA is a powerful systematic biological technique aimed at constructing a co-expression network based on the genes with consistently expressed relationships, further *in vivo*/*in vitro* experiments are required to verify the identified biomarkers.

**Table 4:** Relationship between expression modules with OS within gastric cancer molecular subtypes in the training dataset.

**Modules**	**Gene count**	**MSS/TP53**^−^**(*n* = 107)**			**MSS/TP53**^+^**(*n* = 79)**			**MSI (*n* = 68)**			**MSS/EMT (*n* = 46)**		
		**HR**	**95% CI**	***p*-value**	**HR**	**95% CI**	***p*-value**	**HR**	**95% CI**	***p*-value**	**HR**	**95% CI**	***p*-value**
ME_black_	43	0.839	0.5–1.406	0.504	1.309	0.69–2.484	0.41	0.873	0.376–2.027	0.752	0.759	0.381–1.511	0.433
ME_blue_	404	1.063	0.635–1.78	0.815	0.823	0.435–1.557	0.55	0.779	0.341–1.779	0.553	0.865	0.435–1.72	0.678
ME_brown_	342	1.128	0.674–1.887	0.648	1.403	0.737–2.673	0.303	1.835	0.791–4.257	0.157	1.903	0.949–3.817	0.07
ME_green_	113	1.765	1.043–2.99	0.034^*^	0.961	0.505–1.826	0.903	1.384	0.603–3.177	0.444	0.491	0.241–1.001	0.05^*^
ME_red_	95	0.69	0.411–1.158	0.16	1.512	0.794–2.878	0.209	0.852	0.367–1.975	0.709	1.003	0.505–1.992	0.993
ME_turquoise_	1,059	1.452	0.863–2.443	0.16	0.861	0.456–1.629	0.646	2.174	0.919–5.142	0.077	2.485	1.205–5.124	0.014^*^
ME_yellow_	236	0.82	0.49–1.375	0.452	1.027	0.542–1.945	0.936	0.665	0.287–1.539	0.34	0.63	0.314–1.2	0.193

**Notes.**

* *p* ≤ 0.05.

** *p* ≤ 0.01.

*** *p* ≤ 0.001.

Overall survival (OS). Hazard ratios (HRs), 95% confidence intervals (CI), and *p*-values were calculated using Cox proportional hazards regression analysis after grouped the gastric cancer patients by the median of gene level.

**Table 5:** Relationships between hub genes in module brown with OS within GC molecular subtypes in the training dataset.

**Gene**	**MSS/TP53**^−^**(*n* = 107)**			**MSS/TP53**^+^**(*n* = 79)**			**MSI (*n* = 68)**			**MSS/EMT (*n* = 46)**		
	**HR**	**95% CI**	***p*-value**	**HR**	**95% CI**	***p*-value**	**HR**	**95% CI**	***p*-value**	**HR**	**95% CI**	***p*-value**
COL8A1	0.983	0.587–1.644	0.947	0.967	0.511–1.83	0.918	2.19	0.927–5.176	0.074	2.216	1.104–4.447	0.025^*^
FRMD6	1.092	0.652–1.83	0.738	1.242	0.655–2.356	0.506	2.822	1.156–6.888	0.023^*^	1.562	0.781–3.123	0.207
DDR2	1.208	0.721–2.024	0.473	1.18	0.622–2.239	0.612	1.776	0.767–4.109	0.18	1.934	0.956–3.909	0.066
LOC100505881	1.104	0.66–1.848	0.706	1.336	0.701–2.546	0.379	1.116	0.492–2.531	0.793	1.276	0.642–2.536	0.487
TIMP2	0.965	0.577–1.616	0.893	1.257	0.663–2.382	0.484	2.942	1.199–7.217	0.018^*^	1.704	0.851–3.412	0.132
CNRIP1	1.162	0.693–1.947	0.569	1.217	0.642–2.307	0.548	2.626	1.077–6.401	0.034^*^	1.808	0.902–3.624	0.095
CLEC11A	1.241	0.74–2.08	0.413	1.197	0.633–2.265	0.581	1.732	0.748–4.009	0.2	1.309	0.66–2.597	0.441
MRC2	0.825	0.493–1.381	0.465	1.693	0.882–3.249	0.113	2.27	0.959–5.377	0.062	1.233	0.621–2.449	0.549
BGN	1.295	0.771–2.175	0.329	1.282	0.675–2.438	0.448	2.115	0.895–4.997	0.088	1.816	0.913–3.612	0.089
GPR124	1.017	0.608–1.702	0.948	1.612	0.838–3.103	0.153	3.696	1.451–9.413	0.006^**^	1.536	0.767–3.076	0.226

**Notes.**

* *p* ≤ 0.05.

** *p* ≤ 0.01.

*** *p* ≤ 0.001.

Overall survival (OS). Hazard ratios (HRs), 95% confidence intervals (CI), and *p*-values were calculated using Cox proportional hazards regression analysis after grouped the gastric cancer patients by the median of gene level.

## Conclusion

This study used an effective systematic biology-based WGCNA approach to expose the underlying biological mechanisms and to identify the hub biomarkers (*COL8A1*, *FRMD6*, *TIMP2*, *CNRIP1*, and *GPR124*) suggestive of a GC prognosis. This approach could be applied to personalized therapies. However, large-scale randomized controlled clinical trials and replication experiments are required to evaluate the possible molecular signatures to predict survival and to use these hub genes in a clinical setting.

##  Supplemental Information

10.7717/peerj.8504/supp-1 Figure S1 The heatmap of top 5000 variable genes in the 300 tumor samples

10.7717/peerj.8504/supp-2 Data S1 Summary of gene distribution in the identified WGCNA modules and the results of survival analysis for the 5000 genes in the training dataset and the validating dataset

10.7717/peerj.8504/supp-3 Data S2 Gene ontology analysis of genes in the brown module

10.7717/peerj.8504/supp-4 Data S3 Kyoto Encyclopedia of Genes and Genomes Pathway analysis of genes in the brown module

10.7717/peerj.8504/supp-5 Supplemental Information 1 Relationship between expression modules/hub genes with DFS within gastric cancer molecular subtypes in the training dataset and gene set enriched in gastric samples Relationship between expression modules with DFS within gastric cancer molecular subtypes in the training dataset. Relationships between hub genes in module brown with DFS within GC molecular subtypes in the training dataset. Gene set enriched in gastric samples with COL8A1, FRMD6, TIMP2, CNRIP1 and GPR124 high expression.

## Abbreviations

GC: gastric carcinoma
WGCNA: weighted gene co-expression network analysis
TCGA: The Cancer Genome Atlas
KEGG: Kyoto Encyclopedia of Genes and Genomes Pathway
GO: gene ontology
GSEA: gene set enrichment analysis
OS: overall survival
DFS: disease-free survival
MAD: median absolute deviation

## Additional Information and Declarations

### Data Availability

The following information was supplied regarding data availability: Data is available at: 1. NCBI GEO: GSE62254, GSE34942, GSE15459. 2. The Cancer Genome Atlas Project database (https://cancergenome.nih.gov/)—TCGA-STAD: TCGA Hub-TCGA Stomach Cancer (STAD)-gene expression RNAseq. 3. cBio Cancer Genomics portal: Query-Esophagus/Stomach-Stomach Adenocarcinoma (TCGA, Firehose Legacy)-COL8A1 FRMD6 TIMP2 CNRIP1 ADGRA2. 4. Oncomine (https://www.oncomine.org/resource/login.html): -Analysis Type: Gastric Cancer vs. Normal Analysis-COL8A1; Analysis Type: Gastric Cancer vs. Normal Analysis-FRMD6; Analysis Type: Gastric Cancer vs. Normal Analysis-TIMP2; Analysis Type: Gastric Cancer vs. Normal Analysis-CNRIP1; Analysis Type: Gastric Cancer vs. Normal Analysis-GPR124. 5. The Human Protein Atlas database (https://www.proteinatlas.org/): COL8A1-Tissue-Pathology; FRMD6-Tissue-Pathology; TIMP2-Tissue- Pathology; GPR124-Tissue-Pathology.
