# Supplementary figures and images for: A transcriptional co-expression network-based approach to identify prognostic biomarkers in gastric carcinoma
Source: PeerJ. 2020 Feb 14;8:e8504. doi: 10.7717/peerj.8504 (PMC7025707; doi:10.7717/peerj.8504)

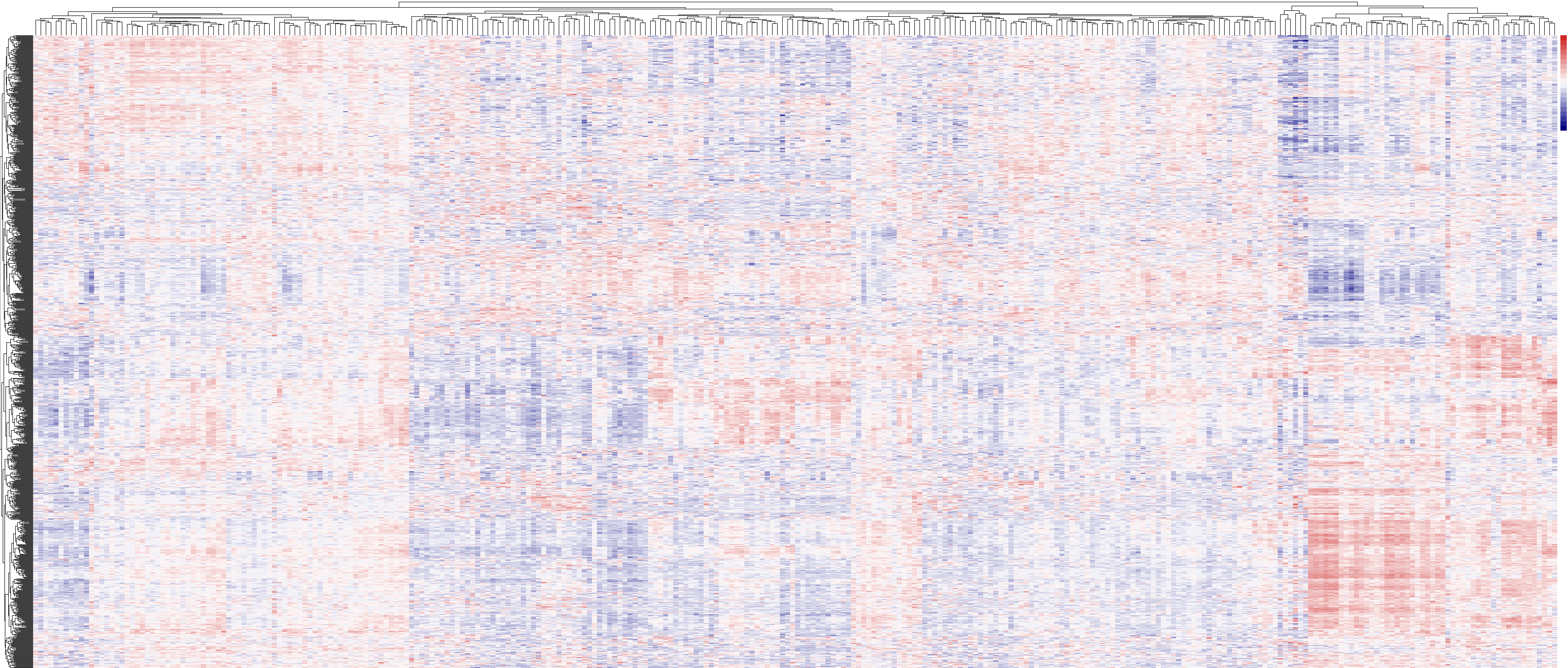

Supplement: Figure S1 [file peerj-08-8504-s001.pdf]
