# Supplementary material for: A transcriptional co-expression network-based approach to identify prognostic biomarkers in gastric carcinoma
Source: PeerJ. 2020 Feb 14;8:e8504. doi: 10.7717/peerj.8504 (PMC7025707; doi:10.7717/peerj.8504)
Supplement: Supplemental Information 1 — Relationship between expression modules with DFS within gastric cancer molecular subtypes in the training dataset. Relationships between hub genes in module brown with DFS within GC molecular subtypes in the training dataset. Gene set enriched in gastric samples with COL8A1, FRMD6, TIMP2, CNRIP1 and GPR124 high expression. [file peerj-08-8504-s005.docx]

**Supplemental Table S1** Relationship between expression modules with DFS within gastric cancer molecular subtypes in the training dataset

| **Modules** | **Gene Count** | **MSS/TP53^-^ (n=107)** | | | **MSS/TP53^+^ (n=79)** | | | **MSI (n=68)** | | | **MSS/EMT (n=46)** | | |
| --- | --- | --- | --- | --- | --- | --- | --- | --- | --- | --- | --- | --- | --- |
|  |  | **HR** | **95%CI** | **p-value** | **HR** | **95%CI** | **p-value** | **HR** | **95%CI** | **p-value** | **HR** | **95%CI** | **p-value** |
| MEblack | 43 | 0.921 | 0.549-1.544 | 0.755 | 1.353 | 0.715-2.56 | 0.353 | 0.649 | 0.284-1.483 | 0.305 | 0.806 | 0.406-1.601 | 0.539 |
| MEblue | 404 | 1.035 | 0.618-1.733 | 0.895 | 0.951 | 0.503-1.799 | 0.877 | 0.821 | 0.36-1.874 | 0.64 | 0.795 | 0.398-1.585 | 0.514 |
| MEbrown | 342 | 0.958 | 0.572-1.604 | 0.87 | 1.454 | 0.763-2.769 | 0.255 | 2.014 | 0.87-4.661 | 0.102 | 1.854 | 0.925-3.715 | 0.082 |
| MEgreen | 113 | 1.545 | 0.912-2.615 | 0.106 | 0.92 | 0.487-1.741 | 0.799 | 1.518 | 0.665-3.466 | 0.321 | 0.512 | 0.252-1.041 | 0.064 |
| MEred | 95 | 0.784 | 0.467-1.316 | 0.357 | 1.528 | 0.805-2.899 | 0.195 | 0.725 | 0.318-1.655 | 0.446 | 0.993 | 0.501-1.969 | 0.984 |
| MEturquoise | 1059 | 1.401 | 0.833-2.357 | 0.204 | 0.79 | 0.418-1.494 | 0.469 | 2.234 | 0.945-5.279 | 0.067 | 2.467 | 1.198-5.081 | 0.014* |
| MEyellow | 236 | 0.876 | 0.523-1.469 | 0.616 | 1.227 | 0.649-2.319 | 0.529 | 0.544 | 0.235-1.258 | 0.155 | 0.661 | 0.33-1.321 | 0.241 |

* *p* ≤ 0.05, ** *p* ≤ 0.01, *** *p* ≤ 0.001. Disease-free survival (DFS). Hazard ratios (HRs), 95% confidence intervals (CI), and p-values were calculated using Cox proportional hazards regression analysis after grouped the gastric cancer patients by the median of gene level.

**Supplemental Table S2** Relationships between hub genes in module brown with DFS within GC molecular subtypes in the training dataset

| **Gene** | **MSS/TP53^-^ (n=107)** | | | **MSS/TP53^+^ (n=79)** | | | **MSI (n=68)** | | | **MSS/EMT (n=46)** | | |
| --- | --- | --- | --- | --- | --- | --- | --- | --- | --- | --- | --- | --- |
|  | **HR** | **95%CI** | **p-value** | **HR** | **95%CI** | **p-value** | **HR** | **95%CI** | **p-value** | **HR** | **95%CI** | **p-value** |
| COL8A1 | 0.971 | 0.58-1.626 | 0.911 | 0.947 | 0.501-1.789 | 0.866 | 2.15 | 0.911-5.074 | 0.081 | 2.295 | 1.144-4.602 | 0.019* |
| FRMD6 | 1.033 | 0.616-1.731 | 0.902 | 1.331 | 0.702-2.523 | 0.382 | 2.715 | 1.116-6.605 | 0.028* | 1.726 | 0.862-3.454 | 0.123 |
| DDR2 | 1.093 | 0.652-1.831 | 0.737 | 1.106 | 0.583-2.097 | 0.758 | 1.928 | 0.833-4.462 | 0.125 | 1.951 | 0.969-3.928 | 0.061 |
| LOC100505881 | 1.119 | 0.669-1.874 | 0.668 | 1.239 | 0.65-2.362 | 0.514 | 1.224 | 0.54-2.775 | 0.629 | 1.295 | 0.652-2.572 | 0.459 |
| TIMP2 | 0.826 | 0.493-1.382 | 0.466 | 1.189 | 0.627-2.254 | 0.597 | 2.964 | 1.217-7.217 | 0.017* | 1.568 | 0.784-3.136 | 0.204 |
| CNRIP1 | 1.03 | 0.615-1.727 | 0.911 | 1.269 | 0.669-2.406 | 0.465 | 2.942 | 1.207-7.168 | 0.018* | 1.636 | 0.817-3.276 | 0.165 |
| CLEC11A | 1.191 | 0.71-1.996 | 0.508 | 1.313 | 0.694-2.485 | 0.402 | 1.755 | 0.759-4.058 | 0.188 | 1.304 | 0.656-2.59 | 0.449 |
| MRC2 | 0.719 | 0.429-1.204 | 0.21 | 1.544 | 0.806-2.96 | 0.191 | 2.379 | 1.007-5.62 | 0.048* | 1.133 | 0.57-2.253 | 0.722 |
| BGN | 1.177 | 0.701-1.976 | 0.538 | 1.451 | 0.765-2.753 | 0.254 | 2.283 | 0.967-5.389 | 0.06 | 1.643 | 0.825-3.272 | 0.158 |
| GPR124 | 0.877 | 0.524-1.468 | 0.618 | 1.61 | 0.839-3.087 | 0.152 | 3.814 | 1.5-9.694 | 0.005** | 1.552 | 0.777-3.102 | 0.213 |

* *p* ≤ 0.05, ** *p* ≤ 0.01, *** *p* ≤ 0.001. Disease-free survival (DFS). Hazard ratios (HRs), 95% confidence intervals (CI), and p-values were calculated using Cox proportional hazards regression analysis after grouped the gastric cancer patients by the median of gene level.

**Supplemental Table S3** Gene set enriched in gastric samples with COL8A1 high expression

| KEGG pathway | ES | NES | NOM p-val | FDR q-val |
| --- | --- | --- | --- | --- |
| Focal adhesion | 0.642164 | 2.010706 | 0 | 0.013562 |
| MAPK signaling pathway | 0.486047 | 1.873360 | 0 | 0.022987 |
| Hypertrophic cardiomyopathy HCM | 0.666637 | 1.842285 | 0 | 0.029878 |
| ECM receptor interaction | 0.780677 | 1.875293 | 0 | 0.032989 |
| Dilated cardiomyopathy | 0.672196 | 1.815344 | 0 | 0.033609 |

ES, enrichment score; NES, normalized enrichment score; NOM p-val, nominal p value; FDR, false discovery rate q value.

**Supplemental Table S4** Gene set enriched in gastric samples with FRMD6 high expression

| KEGG pathway | ES | NES | NOM p-val | FDR q-val |
| --- | --- | --- | --- | --- |
| Hypertrophic cardiomyopathy HCM | 0.676538 | 1.88334 | 0 | 0.025208 |
| Focal adhesion | 0.602799 | 1.89473 | 0 | 0.034539 |
| ECM receptor interaction | 0.756069 | 1.834094 | 0 | 0.035397 |
| Arrhythmogenic right ventricular cardiomyopathy arvc | 0.675432 | 1.813331 | 0.002012 | 0.036139 |
| Dilated cardiomyopathy | 0.701614 | 1.942828 | 0 | 0.040858 |

ES, enrichment score; NES, normalized enrichment score; NOM p-val, nominal p value; FDR, false discovery rate q value.

**Supplemental Table S5** Gene set enriched in gastric samples with TIMP2 high expression

| KEGG pathway | ES | NES | NOM p-val | FDR q-val |
| --- | --- | --- | --- | --- |
| Hypertrophic cardiomyopathy HCM | 0.673980 | 1.911272 | 0 | 0.015696 |
| Dilated cardiomyopathy | 0.688504 | 1.924166 | 0 | 0.021905 |
| Acute myeloid leukemia | 0.578235 | 1.809426 | 0.003724 | 0.024328 |
| Focal adhesion | 0.627413 | 1.963339 | 0 | 0.024577 |
| Chemokine signaling pathway | 0.570614 | 1.788511 | 0 | 0.025981 |
| Arrhythmogenic right ventricular cardiomyopathy arvc | 0.667917 | 1.812366 | 0 | 0.027496 |
| MAPK signaling pathway | 0.466618 | 1.82109 | 0 | 0.029583 |
| ECM receptor interaction | 0.770251 | 1.833021 | 0 | 0.031944 |
| Regulation of actin cytoskeleton | 0.498709 | 1.76472 | 0.001946 | 0.031962 |
| Cell adhesion molecules CAMS | 0.633919 | 1.738564 | 0 | 0.037052 |
| GAP junction | 0.605841 | 1.744520 | 0.001894 | 0.037526 |
| TGF beta signaling pathway | 0.677915 | 1.727839 | 0 | 0.038904 |
| Axon guidance | 0.540830 | 1.698659 | 0.003945 | 0.049397 |

ES, enrichment score; NES, normalized enrichment score; NOM p-val, nominal p value; FDR, false discovery rate q value.

**Supplemental Table S6** Gene set enriched in gastric samples with CNRIP1 high expression

| KEGG pathway | ES | NES | NOM  p-val | FDR  q-val |  |  |
| --- | --- | --- | --- | --- | --- | --- |
| Focal adhesion | 0.568812 | 1.793104 | 0 | 0.105094 |  |  |
| TGF beta signaling pathway | 0.699911 | 1.776705 | 0 | 0.060615 |  |  |
| Hematopoietic cell lineage | 0.731447 | 1.757724 | 0 | 0.060004 |  |  |
| Chemokine signaling pathway | 0.548154 | 1.745753 | 0 | 0.055979 |  |  |
| MAPK signaling pathway | 0.437692 | 1.702496 | 0 | 0.064108 |  |  |
| Long term depression | 0.673645 | 1.682868 | 0 | 0.058043 |  |  |
| Regulation of actin cytoskeleton | 0.477831 | 1.671147 | 0 | 0.061342 |  |  |
| Dilated cardiomyopathy | 0.660609 | 1.814449 | 0.001961 | 0.164772 |  |  |
| Hypertrophic cardiomyopathy HCM | 0.642656 | 1.781195 | 0.001984 | 0.078367 |  |  |
| ECM receptor interaction | 0.716917 | 1.716779 | 0.002037 | 0.059999 |  |  |
| Cell adhesion molecules cams | 0.60306 | 1.694035 | 0.003914 | 0.061828 |  |  |
| Axon guidance | 0.494552 | 1.532844 | 0.00396 | 0.125964 |  |  |
| Arrhythmogenic right ventricular cardiomyopathy arvc | 0.64514 | 1.720169 | 0.003968 | 0.064665 |  |  |
| GAP junction | 0.590007 | 1.686102 | 0.003976 | 0.061453 |  |  |
| Calcium signaling pathway | 0.530217 | 1.636257 | 0.005837 | 0.083227 |  |  |
| Pathways in cancer | 0.389717 | 1.483366 | 0.010267 | 0.162581 |  |  |
| Viral myocarditis | 0.633947 | 1.589857 | 0.012766 | 0.118805 |  |  |
| Melanoma | 0.537271 | 1.548602 | 0.01581 | 0.126685 |  |  |
| Vascular smooth muscle contraction | 0.671363 | 1.589146 | 0.015842 | 0.111859 |  |  |
| FC epsilon R signaling pathway | 0.551342 | 1.584463 | 0.016667 | 0.110042 |  |  |
| Neurotrophin signaling pathway | 0.384245 | 1.541716 | 0.021611 | 0.122107 |  |  |
| FC gamma R mediated phagocytosis | 0.473051 | 1.569696 | 0.021782 | 0.11031 |  |  |
| Leukocyte transendothelial migration | 0.467239 | 1.432471 | 0.03629 | 0.203465 |  |  |
| JAK stat signaling pathway | 0.461648 | 1.502514 | 0.037182 | 0.153394 |  |  |
| Melanogenesis | 0.387778 | 1.46119 | 0.038306 | 0.174978 |  |  |
| Leishmania infection | 0.659579 | 1.581545 | 0.038697 | 0.106513 |  |  |

ES, enrichment score; NES, normalized enrichment score; NOM p-val, nominal p value; FDR, false discovery rate q value.

**Supplemental Table S7** Gene set enriched in gastric samples with GPR124 high expression

| KEGG pathway | ES | NES | NOM p-val | FDR q-val |
| --- | --- | --- | --- | --- |
| Dilated cardiomyopathy | 0.691109 | 1.912495 | 0.002024 | 0.012468 |
| Hypertrophic cardiomyopathy HCM | 0.669598 | 1.884551 | 0 | 0.015833 |
| MAPK signaling pathway | 0.485364 | 1.918648 | 0 | 0.016693 |
| Focal adhesion | 0.629513 | 1.945361 | 0 | 0.021753 |
| ECM receptor interaction | 0.766736 | 1.851674 | 0 | 0.023054 |
| Acute myeloid leukemia | 0.577098 | 1.81114 | 0.004149 | 0.033171 |
| TGF beta signaling pathway | 0.713854 | 1.797139 | 0 | 0.03375 |

ES, enrichment score; NES, normalized enrichment score; NOM p-val, nominal p value; FDR, false discovery rate q value.
